# Preparation and Anodic Bonding Performance of (PEG)_10_LiClO_4_/NaAlOSiO Solid Electrolyte for Packaging

**DOI:** 10.3390/ijms27093837

**Published:** 2026-04-26

**Authors:** Chao Du, Yali Zhao

**Affiliations:** Department of Materials Science and Engineering, Jinzhong University, Jinzhong 030619, China; zhaoyali@163.com

**Keywords:** PEG, solid electrolyte, anodic bonding, tensile properties, electronic packaging

## Abstract

In this study, a polyethylene glycol (PEG)-based solid electrolyte composite (PEG)_10_LiClO_4_/NaAlOSiO suitable for anodic bonding packaging was successfully fabricated via a combined ball milling and hot pressing process. The micromorphology, ion transport characteristics, and mechanical packaging properties of the composite were systematically investigated using characterization techniques including electrochemical impedance spectroscopy, X-ray diffraction, scanning electron microscopy, and anodic bonding performance tests. The results demonstrate that doping with NaAlOSiO molecular sieve can effectively reduce the crystallinity of the polymer matrix, construct more efficient carrier transport pathways, and simultaneously enhance the ionic conductivity and mechanical properties of the material. When the mass fraction of NaAlOSiO doping is 8 wt.%, the composite exhibits a room temperature ionic conductivity of up to 1.31 × 10^−5^ S·cm^−1^. Under room temperature and a bonding voltage of 800 V, the sample with this doping ratio achieves the optimal anodic bonding with metallic Al, and the tensile strength of the bonding interface reaches 5.93 MPa, showing excellent application prospects in micro–nano-packaging.

## 1. Introduction

In recent years, electronic devices have been gradually evolving toward intelligence, integration, miniaturization, and flexibility [1,2,3]. The industrialization of electronic devices is inseparable from the packaging process, where high-quality packaging acts as a critical guarantee for the stable operation of electronic devices. However, the high packaging cost has become one of the main constraints hindering their sustainable development [4,5,6,7,8]. During the packaging of electronic devices, packaging materials and packaging technologies have long been the focus of researchers’ investigations [9,10,11].

Commonly used packaging materials include metals, ceramics (glasses), polymeric solid electrolytes, and other alternatives. Solid electrolyte packaging materials exhibit distinct advantages such as light weight, corrosion resistance, excellent processability, and low cost, making them suitable for large-scale industrial production [12,13,14,15]. Polyethylene glycol (PEG) possesses a favorable spatial coordination structure and high-density electron groups. Composed of flexible polyether segments, its ethoxy groups are prone to conformational transformation and facilitate the formation of a homogeneous system [16,17,18]. Nano-sized Na_56_[(AlO_2_)_56_(SiO_2_)_136_] molecular sieve (NaAlOSiO molecular sieve) features high surface energy, short pore channels, and a large external surface area. Consequently, it is more conducive to the molecular-level combination between the polymer matrix and the molecular sieve, effectively avoiding the agglomeration effect that frequently occurs in conventional inorganic oxide doping [19,20,21,22].

Commonly employed packaging technologies include adhesive bonding, fusion bonding and anodic bonding [9,23,24]. Adhesive bonding realizes material connection via binders, yet it tends to cause device contamination during the operation. In addition, the air-drying of excess binders alters the original dimensions of devices, which is detrimental to miniature devices. Fusion bonding delivers superior packaging quality, but it is accompanied by complicated procedures, low packaging efficiency and relatively high bonding temperature that induces considerable bonding stress; furthermore, the high temperature may also damage the devices. In contrast, anodic bonding boasts prominent merits: low processing temperature, high bonding efficiency, fast bonding speed, excellent sealing performance and low cost. Additionally, it enables direct bonding of functional materials such as metals and ceramics without any auxiliary materials.

In our preliminary work, we demonstrated that the addition of SiO_2_ and CeO_2_ enhances the anode bonding behavior of the (PEG)_10_LiClO_4_ system [9,12]. In this work, NaAlOSiO molecular sieve was employed to modify (PEG)_10_LiClO_4_, and a novel PEG-based solid polymer electrolyte was fabricated as an anodic bonding packaging material. The anode bonding process imposes specific requirements on the cathode materials to be bonded, including excellent ionic conductivity, mechanical stability, and favorable thermal properties. These characteristics serve as critical indicators for evaluating the anode bonding performance of cathode materials. The anodic bonding performance of the as-prepared composite electrolyte was systematically investigated, which provides a new insight into the application of solid electrolytes in the micro–nano-packaging field.

## 2. Results and Discussion

### 2.1. Surface Characterization

**Figure 1:**
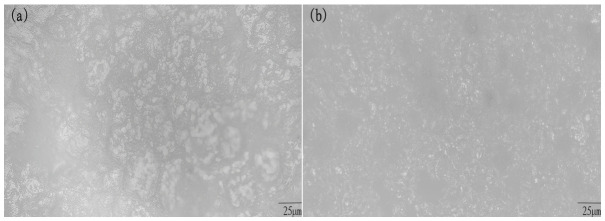

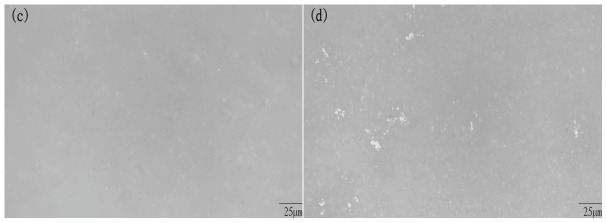
The SEM images of (PEG)_10_LiClO_4_/*x*%wt.NaAlOSiO. (**a**) *x* = 0, (**b**) *x* = 5, (**c**) *x* = 8, (**d**) *x* = 10.

Figure 1 shows the surface morphology of the as-prepared material (PEG)_10_LiClO_4_/*x*%wt.NaAlOSiO. It can be seen that (PEG)_10_LiClO_4_ exhibits obvious spherical crystal characteristics with relatively regular crystallization, which is continuously distributed on the matrix surface. Such a structure is not conducive to ion migration.

After the introduction of NaAlOSiO zeolite into the matrix, the surface crystallization behavior of the composite is significantly modified. It can be clearly observed that the growth of spherical crystals is effectively restricted, accompanied by a remarkable reduction in both crystal size and quantity. When the NaAlOSiO content reaches 8 wt.%, the matrix surface becomes relatively uniform and smooth, with no large-sized spherulites observed. This result demonstrates that NaAlOSiO is sufficiently compatible with (PEG)_10_LiClO_4_, and the crystallization of the polymer matrix is greatly suppressed. Nevertheless, when the NaAlOSiO content is further increased to 10 wt.%, an inhomogeneous stacked morphology emerges on the sample surface, which is attributed to the agglomeration of excess zeolite particles.

### 2.2. X-Ray Diffraction Analysis

**Figure 2:**
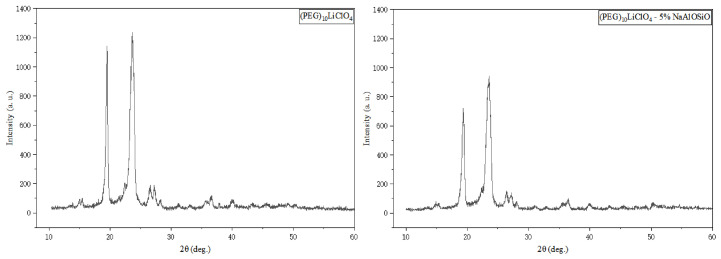

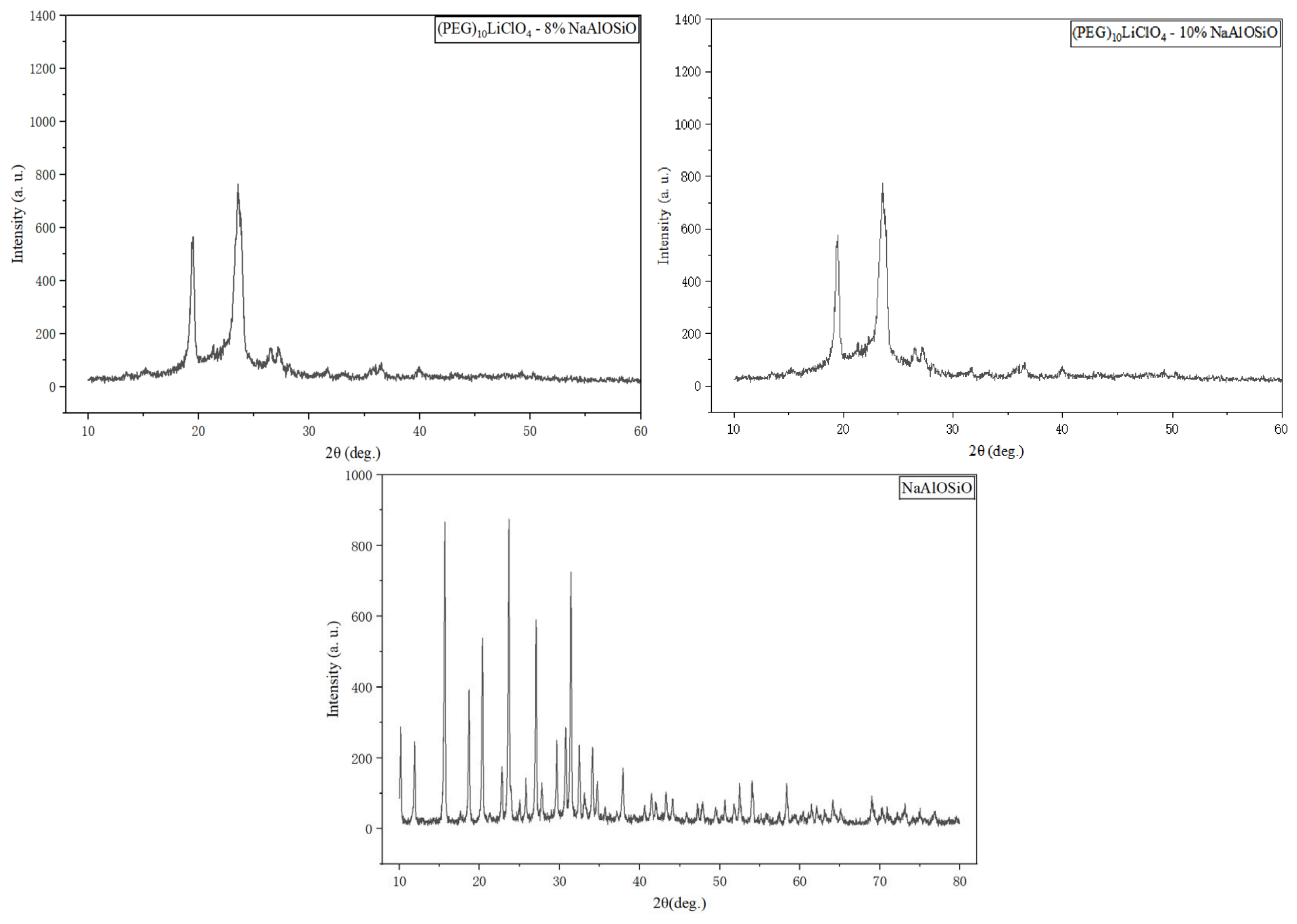
The XRD patterns of Pure NaAlOSiO and (PEG)_10_LiClO_4_/NaAlOSiO.

The crystallinity of the packaging material affects the ion transport efficiency, thereby further influencing the bonding and packaging performance. Figure 2 presents the XRD patterns of the as-prepared solid electrolyte, and the crystallinity of the material can be qualitatively determined according to the intensity of the diffraction peaks. It can be seen from the pattern of (PEG)_10_LiClO_4_/NaAlOSiO that all samples show two relatively obvious diffraction peaks near 19.5° and 23.5°, which are assigned to the characteristic peaks of the matrix material. No characteristic peaks of NaAlOSiO were observed in the XRD pattern of (PEG)_10_LiClO_4_/NaAlOSiO, indicating that the NaAlOSiO powder was well incorporated into the matrix.

The high crystallinity within the matrix hinders ion transport, and the intensity of the aforementioned two characteristic peaks decreases significantly with the addition of NaAlOSiO. This phenomenon can be explained as follows: NaAlOSiO is uniformly dispersed in the PEG matrix, destroying the original ordered crystalline structure of the polymer, disturbing the arrangement of molecular chains, and disrupting the regular thermal motion of ordered chain segments. Consequently, the overall energy provided by the thermal motion of chain segments is reduced, resulting in insufficient energy for crystal nucleation and growth. The entire system fails to achieve complete crystallization, which ultimately leads to a remarkable decline in the relative intensity of the two characteristic diffraction peaks.

When the NaAlOSiO content reaches 8 wt.%, the diffraction peak intensity drops to the minimum, and the proportion of amorphous regions in the system increases accordingly. This effectively extends the ion transport channels, making the structure more conducive to the anodic bonding process. However, when the NaAlOSiO content is further increased to 10 wt.%, the diffraction peak intensity shows no obvious fluctuation, indicating that NaAlOSiO is nearly saturated in the matrix when the doping amount exceeds 8 wt.%, and its regulatory effect on the crystallization behavior of the matrix is no longer significant.

### 2.3. AC Impedance

**Figure 3:**
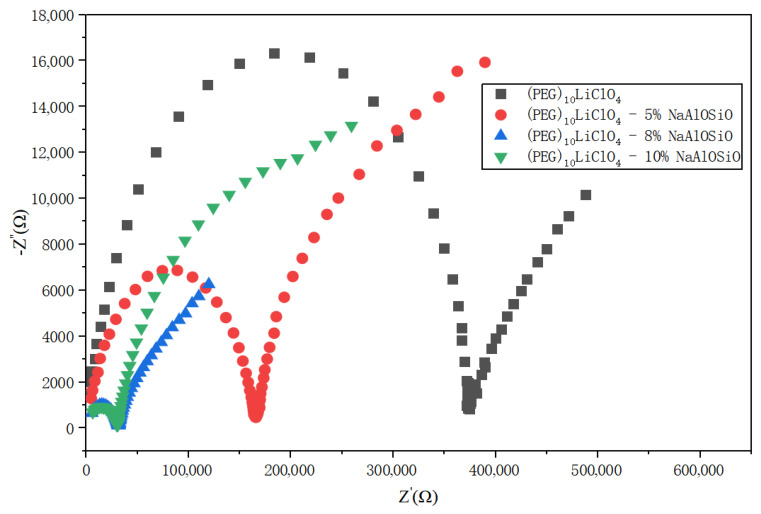
The AC impedance spectra of (PEG)_10_LiClO_4_/NaAlOSiO composite electrolytes at room temperature.

Figure 3 displays the AC impedance plots of the as-prepared (PEG)_10_LiClO_4_/NaAlOSiO composite electrolytes measured at room temperature. As observed, each impedance spectrum consists of two characteristic regions: an incomplete semicircle in the high-frequency zone and a linear tail in the low-frequency zone.

In the pristine sample without NaAlOSiO incorporation, the high-frequency and low-frequency regions are relatively distinguishable, and the bulk resistance (*R*) of (PEG)_10_LiClO_4_ is considerably high. With the gradual increment of NaAlOSiO content, the impedance curves shift toward the high-frequency direction and become increasingly compressed and blurred. The bulk resistance of the composite electrolyte reaches the minimum value at a NaAlOSiO loading of 8 wt.%. Further raising the NaAlOSiO content to 10 wt.% leads to no significant variation in the bulk resistance.

Table 1 presents the ionic conductivity of the as-prepared (PEG)_10_LiClO_4_/NaAlOSiO composite electrolytes tested at room temperature. It is clear that the sample with 8 wt.% NaAlOSiO exhibits the highest room temperature ionic conductivity of 1.31 × 10^−5^ S·cm^−1^. As discussed above, the incorporation of NaAlOSiO can suppress the crystallization of the PEG matrix, thereby expanding the ion transport pathways and improving the ionic conductivity.

At a low NaAlOSiO content (5 wt.%), the ordered structure of the polymer is not sufficiently disrupted, and severe crystallization still exists in local regions of the matrix. When the NaAlOSiO content reaches 8 wt.%, the zeolite uniformly dispersed in the matrix via high-energy ball milling disturbs the arrangement of crystal nucleation structural units, maximizing the disorder degree of the system and increasing the amorphous regions required for ion migration. Furthermore, the large specific surface area of NaAlOSiO creates abundant polymer–filler interfaces, which provide fast transport pathways for Li^+^ ions. The polar surface of the aluminosilicate framework weakens the interaction between Li^+^ and the ether oxygen of PEG, thereby increasing the concentration of mobile charge carriers and improving migration mobility. Further increasing the NaAlOSiO content to 10 wt.% weakens the above modification effects. Excessive additives tend to agglomerate and form ion transport barriers, hindering ion migration and consequently reducing the ionic conductivity of the matrix.

### 2.4. Mechanical Properties Analysis

**Figure 4:**
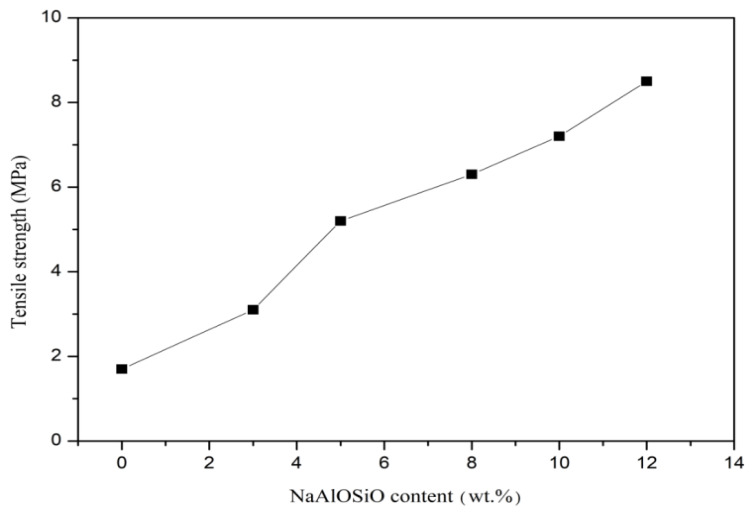
Tensile strength of (PEG)_10_LiClO_4_/NaAlOSiO at room temperature.

Solid electrolytes used as anodic bonding packaging materials also have certain requirements for mechanical properties. During the anodic bonding process, a certain pressure needs to be applied to the bonded materials to ensure tight interfacial contact, which facilitates the smooth progress of the bonding reaction. Figure 4 shows the tensile properties of the as-prepared (PEG)_10_LiClO_4_/NaAlOSiO composite solid electrolytes at room temperature. It can be seen that the tensile strength of the solid electrolytes exhibits an obvious upward trend with the increase in NaAlOSiO content.

This result demonstrates that NaAlOSiO can be well dispersed and exhibits excellent compatibility with the PEG matrix. When subjected to external force, the NaAlOSiO nanoparticles can effectively migrate on the surface and dissipate deformation energy, playing a role in stress dispersion and transfer to a certain extent, thereby ultimately improving the tensile strength of the composite solid polymer electrolyte.

### 2.5. Anodic Bonding Current–Time Curves

**Figure 5:**
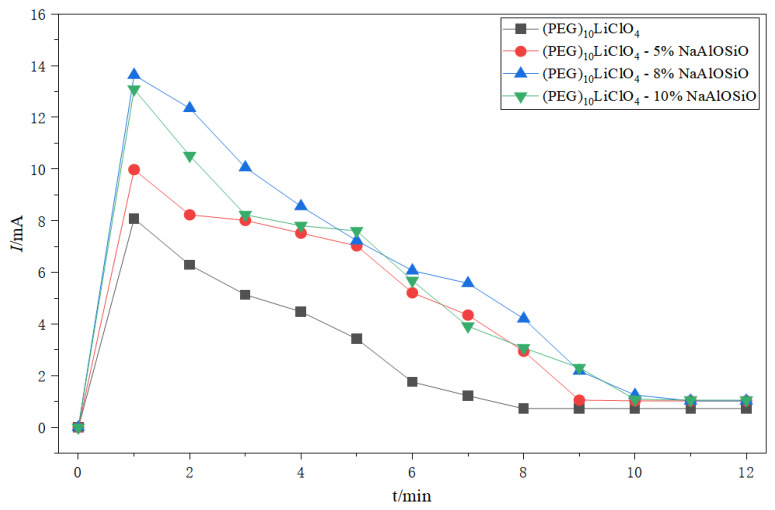
The current–time curves of (PEG)_10_LiClO_4_/NaAlOSiO with Al during anodic bonding.

Figure 5 depicts the time–current curves during the anodic bonding process between the as-prepared (PEG)_10_LiClO_4_/NaAlOSiO composite electrolytes and Al foil at room temperature with a bonding voltage of 800 V. It can be seen that the current of all samples rises rapidly to the maximum value at the initial stage of bonding. As the bonding process proceeds, the bonding current gradually decreases and finally stabilizes at a tiny value. With the incorporation of NaAlOSiO, the peak bonding current changes significantly: the peak current is 10.02 mA at 5 wt.% NaAlOSiO, and reaches the maximum value of 13.96 mA when the content increases to 8 wt.%; further increasing the doping content leads to a decline in the peak current.

During anodic bonding, the current is mainly generated by the effect of an external electrostatic field, which drives the free-moving ions inside the material to migrate directionally, thus forming a current. Therefore, the current value increases rapidly at the initial stage of bonding. As the bonding progresses, ion migration reaches saturation, the interfacial chemical reaction is gradually completed, and the current intensity decreases and stabilizes at a low level, marking the end of the bonding process.

The pristine sample without NaAlOSiO doping has a low peak bonding current of only 8.12 mA, indicating that the number of mobile ions in the system is small, and the high crystallinity of the matrix hinders ion migration. After introducing NaAlOSiO particles, it not only reduces the crystallinity of the system, increases the proportion of amorphous regions, but also releases more free-moving lithium ions, improving the ionic conductivity of the material and thus enhancing the peak bonding current. However, excessive NaAlOSiO cannot continuously increase the peak current, because excessive zeolite particles will agglomerate inside and on the surface of the PEG matrix, forming ion transport barriers and hindering the directional migration of ions.

**Figure 6:**
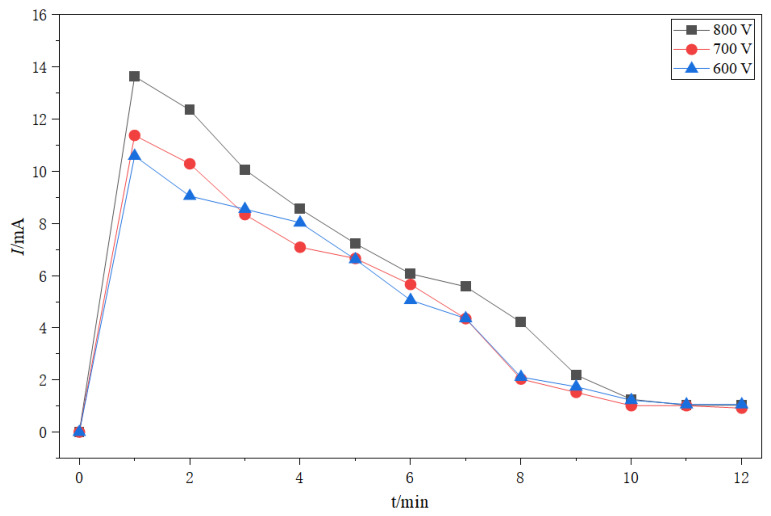
The current–time curve of (PEG)_10_LiClO_4_/8 wt.%NaAlOSiO and Al at different voltages.

Figure 6 presents the time–current curves of anodic bonding between (PEG)_10_LiClO_4_/8 wt.%NaAlOSiO and Al foil at room temperature under various electric field intensities. The variation trend of bonding current for each sample is consistent with the previous regularity. With the decrease in bonding voltage, the peak current of each curve shows an obvious downward trend, suggesting that the electric field intensity exerts a significant regulatory effect on the ion migration rate. When the voltage drops below a critical threshold, the attenuation of peak current intensifies, demonstrating that the driving force for directional ion movement is insufficient to overcome the interfacial barrier and local structural resistance. This phenomenon further confirms that anodic bonding is essentially a synergistic process of ion migration and interfacial reaction driven by electric field.

In addition, increasing the bonding voltage can enhance the electrostatic attraction between bonded materials, promoting tight interfacial contact and accelerating ion migration during bonding, thereby facilitating the bonding reaction and improving the bonding efficiency.

### 2.6. Bonding Interface Analysis

**Figure 7:**
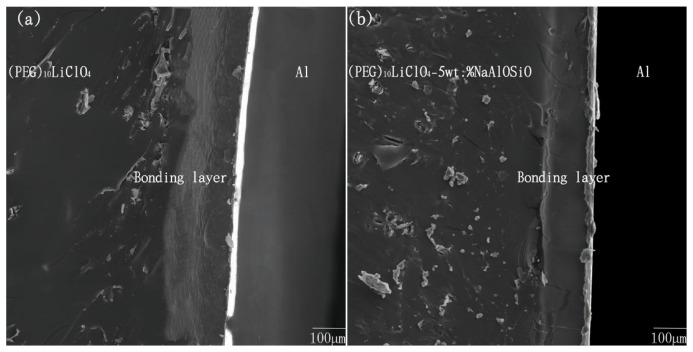

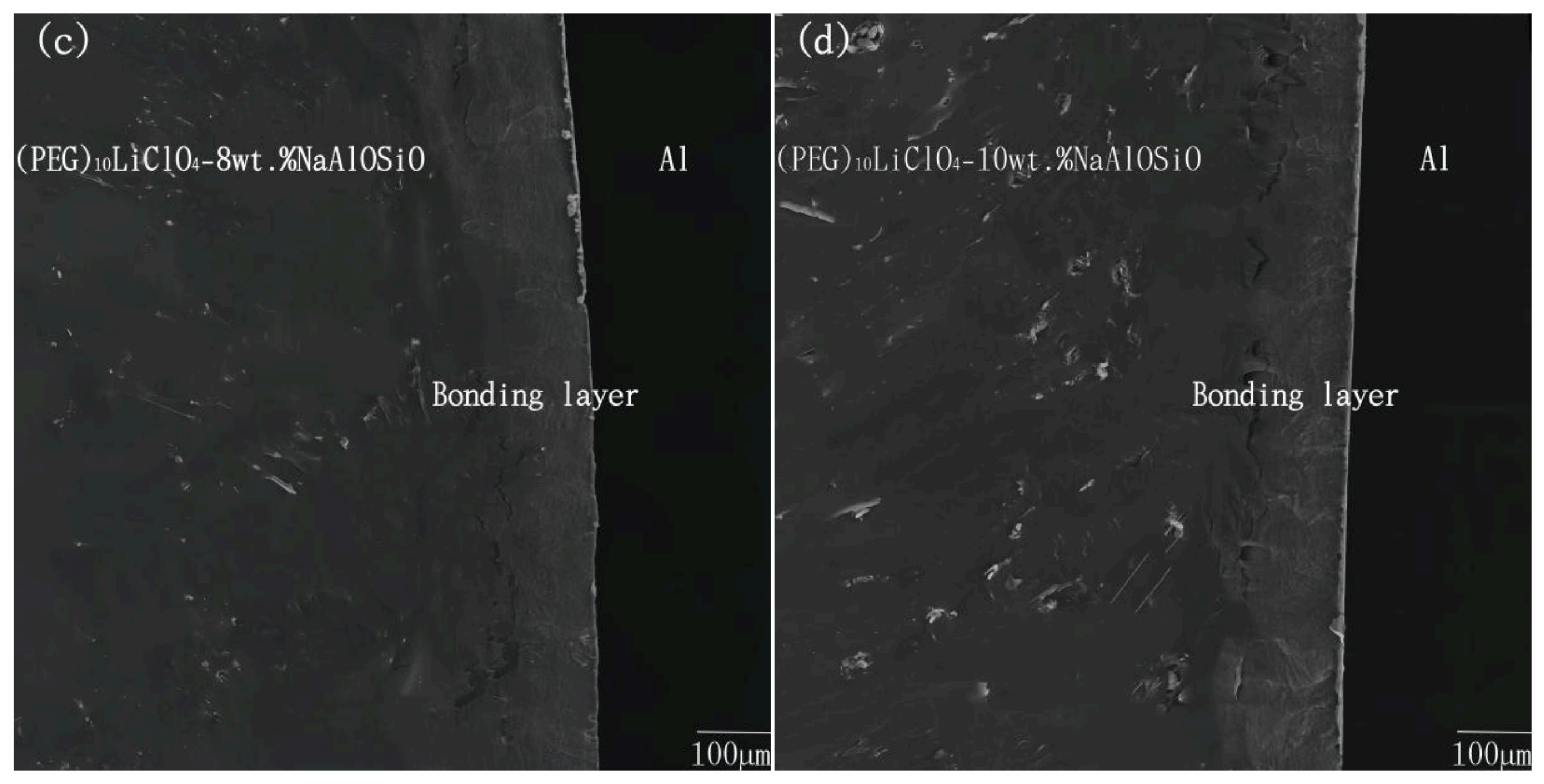
The SEM images of the bonding interface between (PEG)_10_LiClO_4_/*x*%wt.NaAlOSiO and Al. (**a**) *x* = 0, (**b**) *x* = 5, (**c**) *x* = 8, (**d**) *x* = 10.

Figure 7 displays the SEM characterization of the anodic bonding interface between (PEG)_10_LiClO_4_/NaAlOSiO composite electrolytes and Al at room temperature. It can be observed that a distinct bonding layer, which is different from the adjacent matrices, is formed between the as-prepared packaging material and Al substrate. This bonding layer is generated by the ion migration of the packaging material under the synergistic effect of strong electrostatic field and temperature field, followed by complex physicochemical reactions at the interface. When the NaAlOSiO content is 8 wt.%, the intermediate bonding layer exhibits no obvious voids or holes, indicating that the bonding reaction is sufficient and the bonding quality is favorable under this condition.

Table 2 presents the tensile strength of the anodic bonding interface between (PEG)_10_LiClO_4_/NaAlOSiO and Al foil tested at room temperature, and the interfacial tensile strength serves as a critical indicator for evaluating bonding quality. It can be seen from the table that the interfacial bonding strength is improved after the introduction of NaAlOSiO into the PEG matrix. When the NaAlOSiO content is 8 wt.%, the interfacial tensile strength reaches 5.93 MPa, which is 4.06 MPa higher than that of the pristine sample without NaAlOSiO doping.

PEG possesses long polymer chains with weak interchain forces and fragile segments. NaAlOSiO uniformly dispersed in the PEG matrix via high-energy ball milling is embedded between the weak molecular chains, exerting physical cross-linking and reinforcing effects. This not only enhances the mechanical properties of the composite but also optimizes the room temperature ionic conductivity. During the anodic bonding process, more mobile ions can be released from the system, promoting a sufficient bonding reaction and thereby improving the interfacial bonding quality.

## 3. Materials and Methods

### 3.1. Sample Preparation

Polyethylene glycol (PEG) with a molecular weight *M*_w_ = 4000 Da, purity ≥ 99.6%, particle size ≤ 70 μm. Lithium perchlorate (LiClO_4_), analytical grade, particle size < 50 μm. NaAlOSiO (Na_56_[(AlO_2_)_56_(SiO_2_)_136_]) zeolite (NaY), analytical grade, particle size ≤ 300 nm [25].

LiClO_4_ is hygroscopic and hydrated in air quickly. In practical operation, in addition to drying the materials, the following measures should be taken:

Anhydrous LiClO_4_ was stored in an argon-filled glove box with H_2_O < 1 ppm and O_2_ < 1 ppm to prevent contact with air and moisture; All weighing and mixing procedures were performed inside the glove box to minimize exposure to ambient atmosphere; The entire preparation process was carried out rapidly, and samples were immediately sealed after preparation to avoid reabsorption of moisture.

PEG was dried at 50 °C for 48 h, LiClO_4_ was dried at 100 °C for 24 h, and NaY was dried at 120 °C for 3 h. After drying, the powders were mixed in proportion and added into an agate jar. Anhydrous ethanol was used as the grinding agent, and the grinding media consisted of agate balls with diameters of 8, 5, and 3 mm. The experimental ball mill is an XQM-0.5 L planetary ball mill (Changsha Tianchuang Powder Technology Co., Ltd. (Brand: TENCAN), Changsha, China). The ball milling speed was 300 rpm, the milling time was 6 h, and the ball-to-powder mass ratio was 8:1. After ball milling, the fine mixed powder was sieved. The uniformly mixed powder was then heated to a molten state (60 °C), poured into a mold, and pressed into shape. Finally, a circular material with a diameter of 25 mm and a thickness of 2 mm was obtained. The as-prepared (PEG)_10_LiClO_4_/NaAlOSiO (molar ratio: [EO]:[Li^+^] = 10:1)was placed in a drying oven for later use. The water content was 153 ppm for (PEG)_10_LiClO_4_, 122 ppm for (PEG)_10_LiClO_4_/5 wt.% NaAlOSiO, 124 ppm for (PEG)_10_LiClO_4_/8 wt.% NaAlOSiO, and 137 ppm for (PEG)_10_LiClO_4_/10 wt.% NaAlOSiO, respectively. All samples exhibited low water contents below 200 ppm.

### 3.2. Sample Characterization

X-ray diffraction (XRD) analysis was performed on the as-prepared PEG-based solid electrolyte. The instrument used was a Panalytical X’Pert PRO X-ray diffractometer (Malvern Panalytical Ltd. (formerly PANalytical), Almelo, The Netherlands). Before the test, the sample was positioned and placed flat. The main parameters were as follows: Cu-Kα radiation, λ = 0.15406 nm, tube current = 20 mA, tube voltage = 30 kV, scanning rate = 5°/min (2θ), scanning range = 10–60° (2θ).

Electrochemical impedance spectroscopy (EIS) was carried out using a CHI604C electrochemical workstation. The as-prepared (PEG)_10_LiClO_4_/NaAlOSiO was sandwiched between two stainless steel (SS) blocking electrodes. The test was performed at room temperature with a frequency range of 1 Hz–100,000 Hz. Electrochemical impedance spectroscopy (EIS) measurements were carried out using an AC voltage amplitude of 0.02 V. The bulk resistance and ionic conductivity of the sample were obtained from EIS measurements.

The morphology and surface structure of the samples were characterized using a Hitachi S-4800 scanning electron microscope (SEM) (Hitachi High-Technologies Corporation, Tokyo, Japan).

Mechanical properties of the bonded samples were measured using an AP10-10 electro-hydraulic servo fatigue testing machine (Jinan Nake Test Equipment Co., Ltd., Jinan, China). The samples were cut into dimensions of 10 × 10 × 2 mm. The upper and lower surfaces of the sample were bonded to fixtures using AB glue, and the assembly was fixed between the upper and lower clamps of the tensile machine. The setup was stabilized and kept horizontal before testing. Tensile tests were performed at room temperature until the interface was completely separated, with a tensile rate of 0.01 mm/s.

Anodic bonding packaging of (PEG)_10_LiClO_4_/NaAlOSiO with Al were conducted using a JYL/KYJH-1000 anodic bonding platform (Beijing KYKY Technology Co., Ltd., Beijing, China). Aluminum foil was cut and soaked in acetone for 5 min, rinsed with deionized water, then cleaned with a standard RCA solution (NH_4_OH:H_2_O_2_:H_2_O = 0.25:1:5) for 10 min, rinsed again with deionized water, and finally blown dry with nitrogen to avoid re-oxidation.

**Figure 8:**
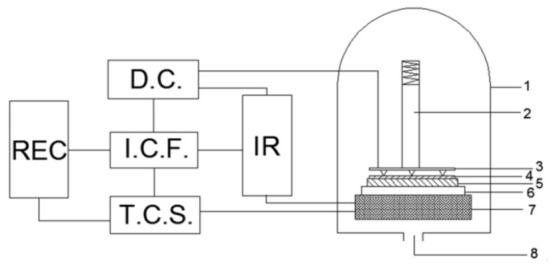
The anodic bonding schematic. 1. Thermal bonding box, 2. Pressurizing pole, 3. Anode, 4. AI, 5. PEG-based solid electrolyte, 6. Cathode, 7. Heating plate, 8. Air. D.C.: direct current mains; IR: data collection system; REC: recording and calculation module; I.C.F.: regulation and protection module; T.C.S.: temperature control system.

The treated Al foil and (PEG)_10_LiClO_4_/NaAlOSiO were stacked and placed into the anodic bonder. Al foil was connected to the anode, and the solid electrolyte to the cathode, as shown in Figure 8. Bonding temperature (room temperature), voltage (600 V–800 V), time (12 min), and pressure (0.2 MPa) were set, and the bonding process was initiated. The bonding current was recorded as a function of time. After bonding, the pressure was maintained, and the sample was furnace-cooled for 1 h at a cooling rate of ~2 °C/min. The sample was then removed, completing the bonding process.

## 4. Conclusions

In this paper, a PEG-based solid electrolyte used as packaging material was fabricated via the high-energy ball milling–hot pressing process, and anodic bonding experiments with aluminum foil were carried out to investigate the anodic bonding performance of the composite. Characterizations including electrochemical impedance spectroscopy, X-ray diffraction, mechanical property and surface morphology analysis reveal that the incorporation of NaAlOSiO zeolite can effectively enhance the mechanical properties of the composite, reduce the crystallinity of the (PEG)_10_LiClO_4_ system and increase the proportion of internal amorphous regions, which facilitates ion migration within the matrix. The composite achieves a room temperature ionic conductivity of 1.31 × 10^−5^ S·cm^−1^ at a NaAlOSiO doping content of 8 wt.%. According to the anodic bonding tests and SEM interfacial characterization, (PEG)_10_LiClO_4_/8 wt.% NaAlOSiO exhibits the highest peak current during anodic bonding with Al, accompanied by a compact and defect-free bonding interface with a tensile strength of 5.93 MPa. In conclusion, NaAlOSiO zeolite can significantly optimize the microstructure, electrochemical and mechanical properties of PEG-based composite solid electrolytes, and effectively improve their anodic bonding performance.

## Figures and Tables

**Table 1:** Ionic conductivity of the as-prepared solid polymer electrolytes (PEG)_10_LiClO_4_/*x*%NaAlOSiO at room temperature.

NaAlOSiO Content (wt.%)	Thickness (cm)	Electrode–Electrolyte Contact Area (cm^2^)	Bulk Resistance (Ω)	Ionic Conductivity (S·cm^−1^)
0	0.2	0.5	3.72 × 10^5^	1.08 × 10^−6^
5%	0.2	0.5	1.69 × 10^5^	2.34 × 10^−6^
8%	0.21	0.5	3.21 × 10^4^	1.31 × 10^−5^
10%	0.19	0.5	3.23 × 10^4^	1.18 × 10^−5^

**Table 2:** Tensile strength of the bonding interface between (PEG)_10_LiClO_4_/NaAlOSiO and Al at room temperature.

NaAlOSiO Content (wt.%)	Area (mm^2^)	Load (N)	Tensile Strength (MPa)
0	100	187	1.87
5	100	465	4.65
8	100	593	5.93
10	100	526	5.26

## Data Availability

The original contributions presented in this study are included in the article. Further inquiries can be directed to the corresponding author.

## References

[1] Bender E., Sitbon M., Avraham T., Gerasimov M. (2025). Spatial Monitoring of I/O Interconnection Nets in Flip-Chip Packages. Electronics.

[2] Wang C., Lin Q., Pan Z., Hong J., Zhou Y. (2024). Thermal contact analysis of Flip-Chip package considering microscopic contacts of double-layer thermal interface materials. Appl. Energy.

[3] Elhadad A., Gao Y., Choi S. (2023). Integrating Renewable Microbial Fuel Cells in Dual In-Line Package for Chip-On-Board Circuits. Adv. Mater. Technol..

[4] Malik M.H., Grosso G., Zangl H., Binder A., Roshanghias A. (2021). Flip Chip integration of ultra-thinned dies in low-cost flexible printed electronics; the effects of die thickness, encapsulation and conductive adhesives. Microelectron. Reliab..

[5] Samotaev N.N., Oblov K.Y., Gorshkova A.V., Ivanova A.V., Philipchuk D.V. (2020). Ceramic packages prototyping for electronic components by using laser micromilling technology. J. Phys. Conf. Ser..

[6] Ghosh U., Shen L.Y. (2026). Effective thermo-mechanical properties deformation characteristics of through-glass-via (TGV) structures in microelectronic packaging. Microelectron. Reliab..

[7] Lei S., Tian D., Wang J., Cui J., Duan Z., Xu Y., Hu Y. (2026). Two-in-one demonstration of anisotropic thermal management near-field EMI shielding via vertically aligned carbon fibers composites in electronic packaging. Compos. Part B.

[8] Alharshan G.A., Shaaban S.M., Said S.A., Elsad R.A., Elsafi M. (2025). Gamma-Attenuation, Dielectric Behavior, and Structure of Nd^3+^-Modified Borosilicate Glasses: A Potential Electronic Encapsulation Material. J. Electron. Mater..

[9] Gupta K., Sharma A., Borse K. (2026). Thermal and dielectric properties of blast furnace dust-reinforced composite materials for electronic packaging applications. Mater. Lett..

[10] Wang X., Yang Z., Bian L., Liu W., Zhang G., Zhang J., Chen C., Liu P. (2025). Copper-based composite sintering materials and reliability analysis for power electronics packaging. J. Sci. Adv. Mater. Devices.

[11] Hu Z., Zhang B., Yu H., Lei Y., Xiong H., Jiang B., Chen D. (2025). Research Progress of Interfacial Modification of Copper/Diamond Composites for Electronic Packaging. Int. J. Thermophys..

[12] Du C., Liu C.-R., Yin X. (2017). Polyethylene Glycol-Based Solid Polymer Electrolytes: Encapsulation Materials with Excellent Anodic Bonding Performance. J. Inorg. Organomet. Polym. Mater..

[13] Polat Y. (2026). High conductivity and thermal robust bismuth oxide electrolytes Co-doped with samarium, europium, and cerium for intermediate-temperature solid oxide fuel cell applications. J. Solid State Chem..

[14] Choi N., Kim S., Song S.-W., Roh K. (2026). Electrochemical modeling of capacity fade in lithium metal batteries: Effects of solid electrolyte interphase formation and dead lithium. J. Power Sources.

[15] Chi F., Shen J., Liu S., Pan H., Quan H., Zhu S. (2026). Fluorination of PEO-based polymer electrolytes and their application in solid-state lithium batteries. Adv. Nanocomposites.

[16] Zhang L., Liang Z., Cao X., Chen Y., Zhang D. (2025). Effects of Polyethylene Glycol Molecular Weight and Content on the Properties of PEG-Block-Modified Epoxy Resins. J. Appl. Polym. Sci..

[17] Chang Y., Wang Z., Liu F. (2025). Tunable Crosslinked Polyvinyl Alcohol/Polyethylene Glycol (cPVA/PEG) Nanofiber Membranes with Enhanced Mechanical and Hydrophilic Balance. Molecules.

[18] Ye X., Wang C., Wang L., Lu B., Gao F., Shao D. (2022). DLP Printing of a Flexible Micropattern Si/PEDOT:PSS/PEG Electrode for Lithium-Ion Batteries. Chem. Commun..

[19] Zhang K., Duan X., Chen A., Liu Y., Chao Y. (2026). Synergistic pore modulation surface engineering of NaYmolecular sieves by N-CQDs for high-efficiency CO_2_ adsorption. Solid State Sci..

[20] Rostami S., Pour A.N., Mohammadi A. (2025). The Effect of Competitor Molecules in the Adsorptive Desulfurization of Sulfurous Organic Solution by the Ni-NaY Zeolite Adsorbent. Chem. Afr..

[21] Hosseinpour E., Rahbar-Kelishami A. (2025). Cationic surfactant modified NaY zeolite: Preparation, investigating the effect of surfactant concentration, and application for methyl orange adsorption from aqueous solution. J. Environ. Chem. Eng..

[22] Si W., Hu W., Wang W., Zhang X., Zhao X., Ma Z., Wang Y., Xing C. (2025). Dual-bed configuration with eco-friendly NaY synthesis promotes isoparaffin formation from syngas. Microporous Mesoporous Mater..

[23] An N., Li B., Wang Y., Sun X., He X. (2026). An improved unified creep-plasticity constitutive model for viscoplastic solder materials of electronic packaging subjected to high-strain-rate impact loadings. J. Mater. Sci. Mater. Electron..

[24] Wei Z., Yuan Y., Wang Y.-X. (2025). Polymer Materials for Stretchable Electronics Encapsulation. Chem. Res. Chin. Univ..

[25] Du C., Liu C., Yin X., Zhao H. (2021). Effect of rare earth oxide CeO_2_ on the anodic bonding performance of PEG-based MEMS encapsulation materials. Adv. Mech. Eng..

